# Medication Blisters: A Rare Cause of Bowel Perforation

**DOI:** 10.7759/cureus.77751

**Published:** 2025-01-20

**Authors:** Lachezar Lalov, Nicolas Naccarella, Jacques Rommens

**Affiliations:** 1 Department of Radiology, CHIREC Delta, Brussels, BEL; 2 Department of Medicine, Université Libre de Bruxelles, Brussels, BEL

**Keywords:** bowel perforation, computed tomography, foreign-body, multiplanar reconstruction, volume rendering technique

## Abstract

Foreign body ingestion is a common occurrence in vulnerable populations, predominantly at the ends of the age spectrum. While most cases are uncomplicated, some patients may require endoscopic or surgical intervention to prevent serious complications. Therefore, accurate and detailed radiological evaluation is essential for therapeutic decision-making.

We present the case of an 80-year-old woman presenting with abdominal pain and radiological evidence of a foreign body in the small intestine. A conservative treatment approach was initially proposed due to the absence of complications and the hypothesis that a fishbone was the cause. However, her condition deteriorated due to bowel injury, complicated by bleeding and perforation. Multiplanar and 3D reconstructions identified the object as a medication blister located in the sigmoid colon. Urgent surgical intervention allowed for the retrieval of the object and the closure of the bowel perforation. This case highlights the importance of early, detailed, and accurate radiological evaluation to identify the characteristics of foreign bodies and guide timely intervention.

## Introduction

Ingested foreign bodies are not uncommon, particularly in older adults, children, and individuals with psychiatric conditions. Most foreign bodies (80-90%) pass through the gastrointestinal (GI) tract without complications; however, approximately 10-20% require endoscopic removal, and less than 1% result in complications such as perforation [1,2]. The terminal ileum, cecum, and rectosigmoid junction are the most common sites of lower GI perforation, likely due to acute angulation and narrowing of the intestinal lumen​ [1]. Ingesting medication blisters is a rarer form of foreign body ingestion, typically observed in elderly patients, particularly those with cognitive impairments or polypharmacy [1,3,4].

Radiologic evaluation, including advanced techniques such as computed tomography (CT) multiplanar reconstructions (MPR) and 3D volume rendering (VRT), is crucial in identifying the true nature of the foreign body and preventing serious complications [1,4].

## Case presentation

**Figure 1:**
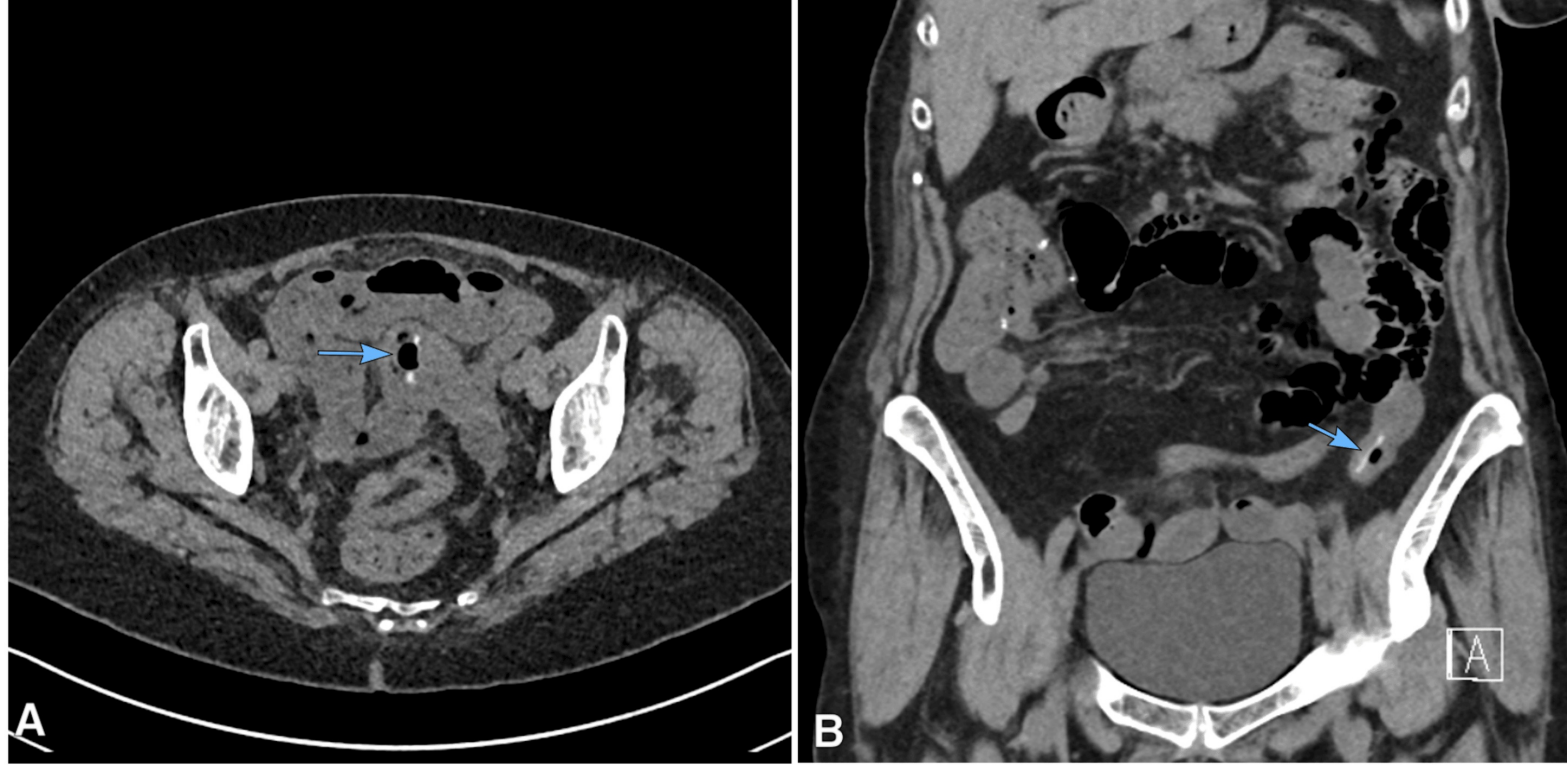
Non-enhanced abdominal CT scan at the time of initial presentation A spontaneously hyperdense structure (blue arrow) is seen within the ileum in the lower left quadrant. Axial view (A); coronal view (B).

An 80-year-old woman with a history of right hemicolectomy for colon adenocarcinoma presented to the emergency department with complaints of lower abdominal pain. On physical examination, tenderness was noted in the affected region. Laboratory findings revealed elevated C-reactive protein (CRP) levels. An abdominal CT scan was performed as part of the evaluation, revealing a linear, hyperdense foreign body, measuring approximately 23 mm, within the ileal lumen in the lower left quadrant (Figure 1). At the time, the possibility of a fishbone was suggested, and due to the patient's stable condition, conservative management with close monitoring was initiated.

**Figure 2:**
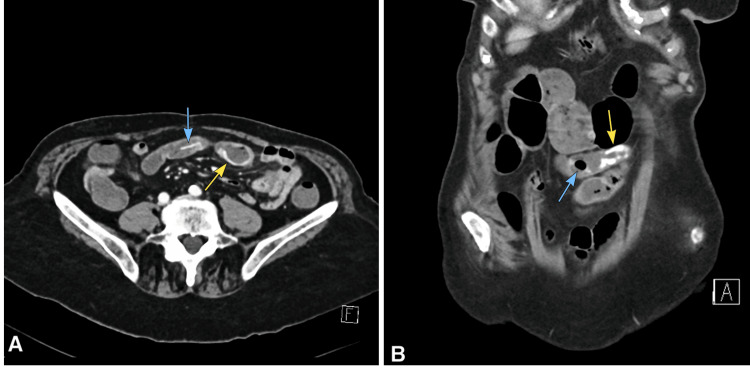
Portal venous phase contrast-enhanced abdominal CT scan at the time of the first follow-up Axial (A) and coronal (B) images showing the linear hyperdense structure (blue arrow) in the terminal ileum. Contrast extravasation is seen at the bowel wall, consistent with the source of bleeding (yellow arrow).

Over the following days, her symptoms persisted and were further complicated by the appearance of blood in her stool. A multiphasic CT scan was ordered to reassess the position of the foreign body and evaluate for potential GI bleeding (Figure 2). The results showed that it was situated in the terminal ileum, near a region of contrast extravasation originating from the intestinal wall, representing the source of bleeding. Given the location, it was believed that the object could be visualized and extracted during a colonoscopy. However, the procedure failed to locate the foreign body.

**Figure 3:**
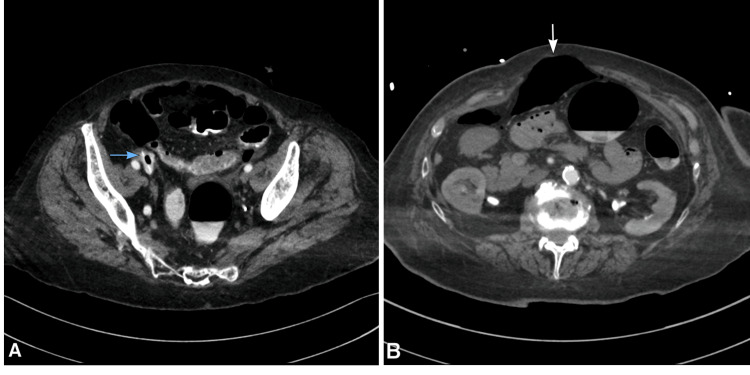
Portal venous phase contrast-enhanced abdominal CT scan, following the colonoscopy procedure The previously described foreign body is situated in the sigmoid colon (A). Extensive extra-visceral gas (white arrow) is observed, suggestive of bowel perforation (B).

**Figure 4:**
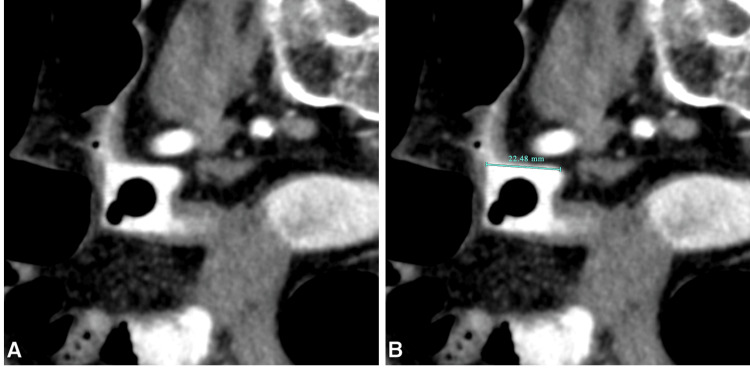
Multiplanar reconstruction in the plane of the foreign body A square-shaped structure with a central round gas-density area is observed (A). Its dimensions are measured at approximately 22.5 mm (B).

**Figure 5:**
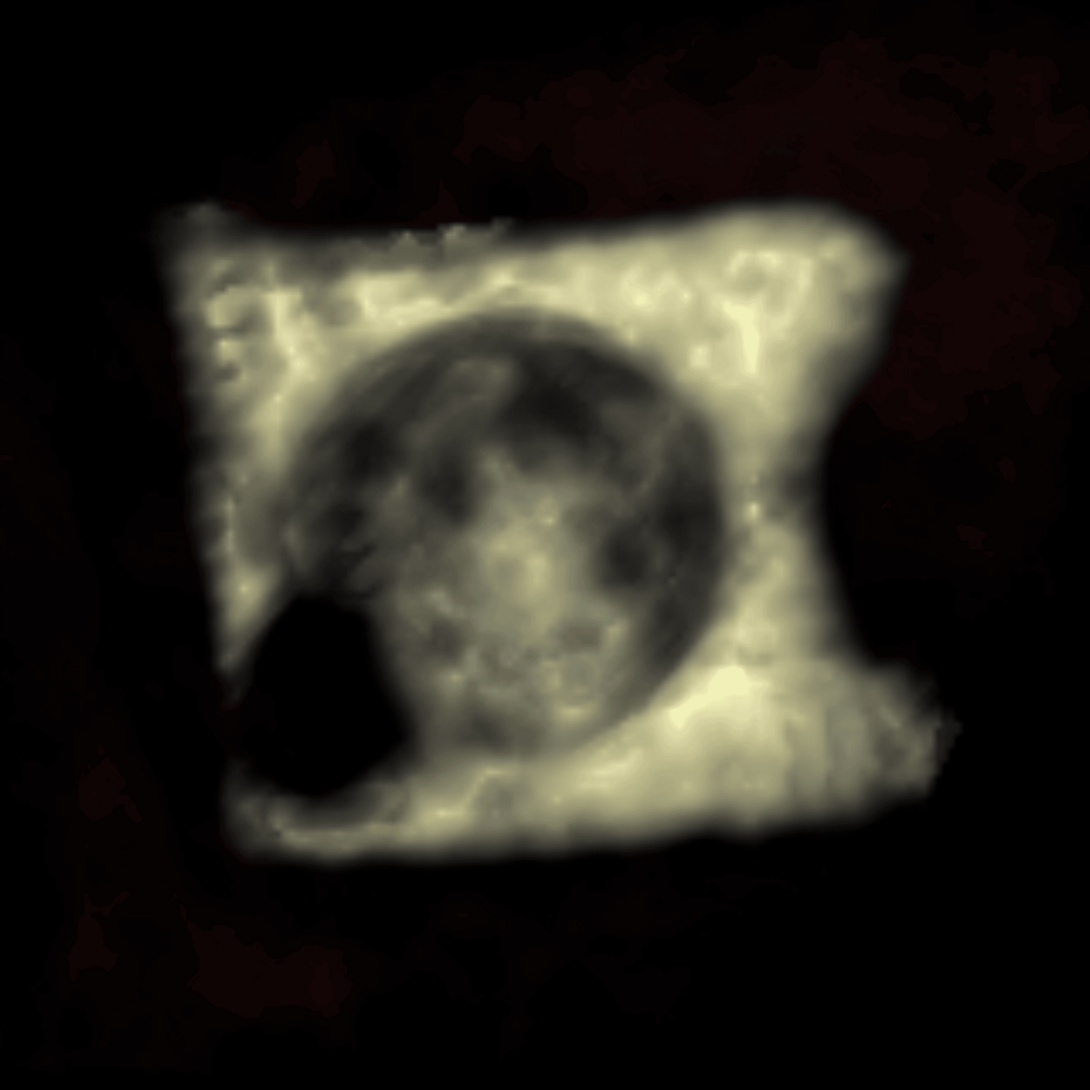
Virtual rendering technique reconstruction of the foreign body A volume rendering reconstruction revealing a thin, square-shaped structure with a central spherical gas bubble.

In the hours following the colonoscopy, the patient's condition deteriorated rapidly, with increasing pain and a significant drop in hemoglobin levels. An urgent repeat CT scan identified the foreign body, located in the sigmoid colon (Figure 3A), associated with extensive extraluminal gas in the peritoneal cavity, indicative of bowel perforation (Figure 3B). To better characterize the object, MPR and VRT reconstructions were performed (Figures 4-5), revealing a thin, square-shaped, hyperdense object measuring approximately 23 x 23 mm, with a central circular gas bubble, consistent with a non-organic foreign body.

**Figure 6:**
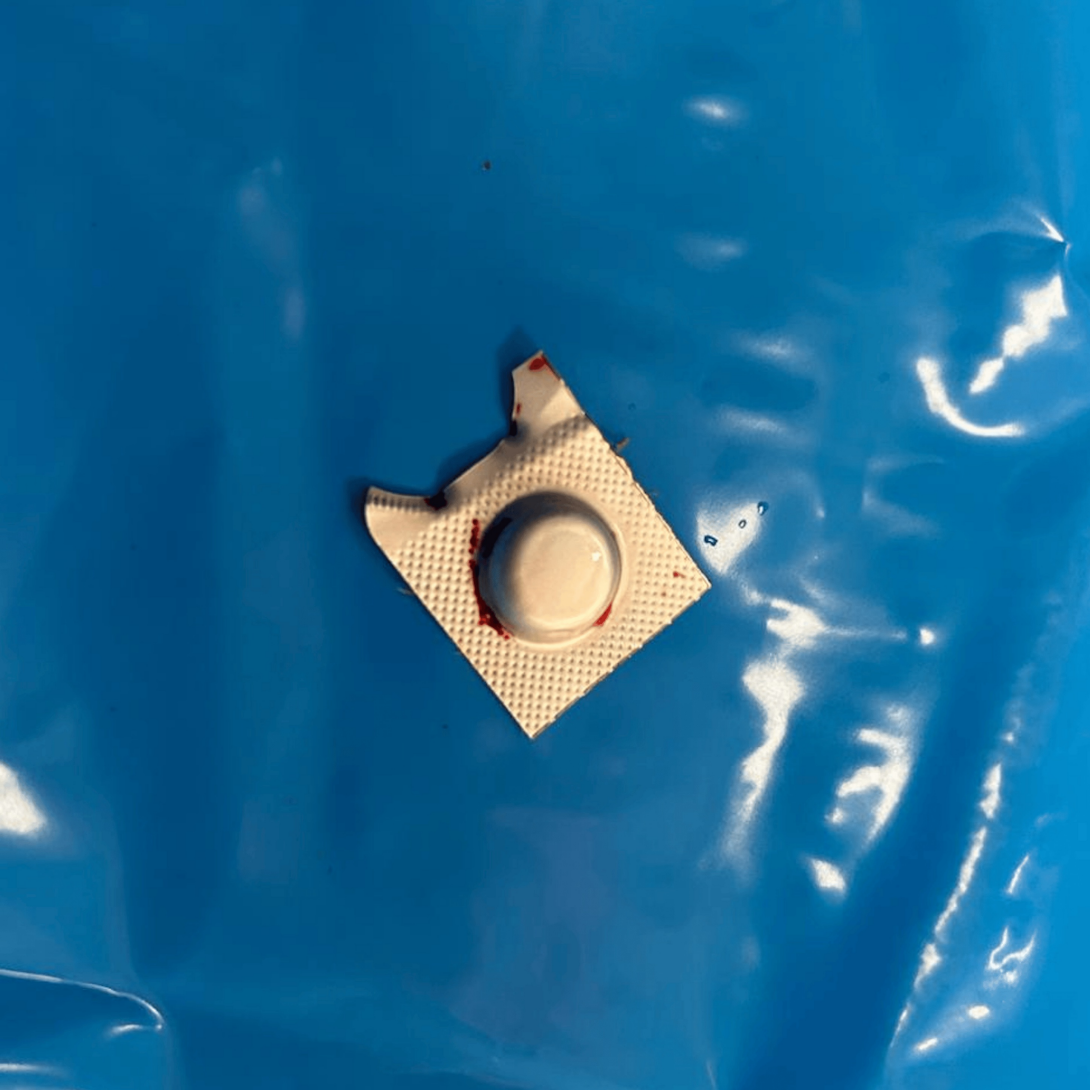
Perioperative photograph of the foreign body: an unopened medication blister

The patient was immediately taken to the operating room for exploratory surgery. During the procedure, the surgeon successfully retrieved a medication blister (Figure 6), with the pill still intact inside. The perforation was repaired, and a thorough abdominal lavage was performed. The patient remained hospitalized for postoperative care and was discharged after making a full recovery.

## Discussion

We present the case of a patient with lower GI bleeding and bowel perforation as the result of a foreign body ingestion, a rare but serious complication. Although visualized, the object’s exact nature was initially overlooked, likely due to the absence of serious complications at the time of initial evaluation. The fishbone hypothesis led to the decision for conservative management, allowing for complications to develop.

This case highlights the importance of thorough radiological evaluation in identifying the characteristics of ingested foreign bodies, including shape, size, composition, and localization, to select the most appropriate course of therapeutic action. CT is a highly useful and widely available tool for evaluating ingested foreign bodies. It provides detailed visualization of anatomical structures and complications, such as perforation, bleeding, or abscess formation, offering greater diagnostic accuracy compared to plain radiographs. However, identifying the foreign body depends on its composition (density) and location [5,6]. The ability to perform multiplanar evaluations makes CT especially valuable in complex cases, like this one, where a sharp-edged object presents a significant risk of perforation [5,7].

The literature suggests that serious complications from foreign body ingestion, including perforation, occur in approximately 1% of cases [1,2,4]. However, in cases of sharp objects, such as medication blisters, the risk of perforation is significantly higher [5,6]. These sharp-edged blisters are especially dangerous due to the high risk of mucosal injury, leading to perforation or impaction [4].

CT imaging should not only be used to localize the foreign body but also to assess its shape, size, and material properties, which directly impact management decisions. For instance, large or sharp foreign bodies may require early surgical intervention, as these are unlikely to pass through the GI tract naturally without causing harm [5,8]. Guidelines emphasize the need for urgent intervention for objects larger than 6 cm in length, while objects measuring over 2.5 cm require semi-urgent intervention [5]. Furthermore, identified sharp objects require close follow-up and surgical extraction in case no movement is observed for longer than three days [5]. Prolonged observation may increase the risk of serious complications, including perforation or obstruction [5]. In the case of our patient, observation was initially proposed due to the suspected linear object and its small size (<2.5 cm).

## Conclusions

In conclusion, this case highlights the importance of comprehensive CT evaluation and timely intervention in managing foreign body ingestion, particularly in high-risk cases involving sharp or large objects. Early recognition of such foreign bodies through advanced imaging techniques can guide appropriate therapeutic decisions and help prevent life-threatening complications, as demonstrated in this case.
